# Multisensory-driven facilitation within the peripersonal space is modulated by the expectations about stimulus location on the body

**DOI:** 10.1038/s41598-022-21469-w

**Published:** 2022-11-21

**Authors:** Alice Rossi Sebastiano, Irene Ronga, Carlotta Fossataro, Mattia Galigani, Karol Poles, Francesca Garbarini

**Affiliations:** 1 MANIBUS Lab, Department of Psychology, University of Turin, Via Verdi 10, 10124 Turin, Italy; 2 BIP Research Group, Department of Psychology, University of Turin, Turin, Italy; 3 NIT, Neuroscience Institute of Turin, Turin, Italy

**Keywords:** Perception, Consciousness, Human behaviour

## Abstract

Compelling evidence from human and non-human studies suggests that responses to multisensory events are fastened when stimuli occur within the space surrounding the bodily self (i.e., peripersonal space; PPS). However, some human studies did not find such effect. We propose that these dissonant voices might actually uncover a specific mechanism, modulating PPS boundaries according to sensory regularities. We exploited a visuo-tactile paradigm, wherein participants provided speeded responses to tactile stimuli and rated their perceived intensity while ignoring simultaneous visual stimuli, appearing near the stimulated hand (VTNear) or far from it (VTFar; near the non-stimulated hand). Tactile stimuli could be delivered only to one hand (unilateral task) or to both hands randomly (bilateral task). Results revealed that a space-dependent multisensory enhancement (i.e., faster responses and higher perceived intensity in VTNear than VTFar) was present when highly predictable tactile stimulation induced PPS to be circumscribed around the stimulated hand (unilateral task). Conversely, when stimulus location was unpredictable (bilateral task), participants showed a comparable multisensory enhancement in both bimodal conditions, suggesting a PPS widening to include both hands. We propose that the detection of environmental regularities actively shapes PPS boundaries, thus optimizing the detection and reaction to incoming sensory stimuli.

## Introduction

An optimal interaction with the environment strongly depends upon an efficient coding of the location of external stimuli relative to the position of the bodily self. In other words, the representation of space is not homogenous, but rather defined in terms of distance from the self-body. Sensory events occurring within the space immediately surrounding the body (i.e., peripersonal space; PPS) are particularly relevant for behavior, since they may be crucial to define the space of the bodily self^1^, to perform goal-directed actions^2^ and to detect potential threats^3^. It is not surprising, therefore, that the processing of such stimuli results enhanced when compared to the processing of the same sensory events occurring in the far space^1^. Interestingly, PPS is also characterized by the presence of multisensory integration, since it represents the area where somatosensory stimulation on the body may be integrated with auditory and/or visual events^1^. Auditory/visual stimuli, delivered within the PPS of a specific body district, are able to enhance the responses to a simultaneous somatosensory stimulation occurring on the same district. In nonhuman primates, bimodal neurons responding to both somatosensory and visual/auditory stimuli presented near to, but not far from, the body have been described within the intraparietal sulcus and the ventral premotor cortex^4–7^. In human studies, this space-dependent multisensory integration effect was observed both at the behavioral level, speeding up the response times (RTs) to tactile stimulation^8–14^ and at the neurophysiological level, with magnified electrophysiological responses for the integrated inputs^15–20^. As a consequence, multisensory integration responses are often considered to index the PPS extent, with multisensory-driven facilitations decreasing while the distance between the tactilely-stimulated body district and auditory/visual stimulation increases^1,8,13^. However, some scholars still questioned the idea that multisensory integration responses may reveal the boundaries of the PPS. Although the research presented above seems to confirm the presence of a significant space-dependent modulation of multisensory-driven facilitations, a number of human studies did not find this effect^21–28^. More specifically, these studies found a generalization of multisensory facilitation effects, in all bimodal conditions relative to the unimodal ones, irrespective of the spatial location of the concurrent auditory/visual stimulus (i.e., whether it was close to or far from the stimulated body district).

After a deep investigation of the current literature, we hypothesized that such a heterogeneity in the results, rather than indicating a methodological issue, might instead uncover the presence of a specific mechanism, inducing or preventing the spatial modulation of multisensory integration. We believe we identified one main factor that could regulate this mechanism. We noticed that the studies which reported the space-dependent effect leveraged on multisensory tasks wherein tactile stimuli were always delivered on the same hand, whereas concurrent cross-modal (e.g., auditory/visual) stimuli could appear either near to or far from it (henceforth referred to as *unilateral tasks*). Conversely, studies which failed to replicate the space-dependent effect capitalized on multisensory tasks wherein both tactile and cross-modal stimuli could occur either on one hand or on the other one (henceforth referred to as *bilateral tasks*). Unilateral and bilateral tasks differ substantially because of an important methodological aspect, which in turn might represent the crucial determinant of the space-dependent multisensory effects. By manipulating the probability of the occurrence of tactile stimulation on each hand (i.e., 100% on one single hand in unilateral tasks; 50% on each hand in bilateral tasks), we expected to modulate the contextual saliency of visual stimuli occurring close to either the stimulated or the non-stimulated hand. In particular, we hypothesized that the spatial modulation of multisensory integration would be dependent on the expectation induced by the predictability of tactile stimulation on the involved body parts. When participants have higher expectation to receive tactile stimuli on one hand only (as in unilateral tasks), this would increase the salience of visual stimuli occurring close to the stimulated hand to the detriment of those occurring close to the non-stimulated hand, thus strengthening the space-dependent modulation of multisensory integration. Conversely, in bilateral tasks, when participants have the same expectation to receive tactile stimuli on each hand, visual stimuli occurring close to both stimulated and non-stimulated hands are afforded with equal salience, thus weakening the space-dependent modulation of multisensory integration.

In the present study, we capitalized on a visuo-tactile multisensory (VT) paradigm, wherein tactile stimuli were delivered alone or simultaneously with visual ones, appearing either near to or far from the stimulated hand. Participants were instructed to provide speeded responses (implicit measure) and rate the perceived intensity (explicit measure) of tactile stimuli. They underwent both a unilateral VT task, in which tactile stimuli were always delivered to their left hand, and a bilateral VT task, in which tactile stimuli could occur either on their right or on their left hand with equal probability. Importantly, as in previous studies^21–24,26^, task-irrelevant visual stimuli occurring far from the stimulated hand were presented near to the non-stimulated hand in both tasks, thus excluding that the simple visible presence of the non-stimulated hand could account for any differential result. We anticipated a space-dependent multisensory enhancement in the unilateral task, with faster responses and greater intensity ratings in the bimodal near as compared to the bimodal far condition. Conversely, we expected this space-dependent modulation to be abolished in the bilateral task, with a comparable multisensory enhancement for both bimodal conditions.

## Methods

### Participants

Sample size was estimated with an a priori analysis employing G*Power software (https://www.psychologie.hhu.de/arbeitsgruppen/allgemeine-psychologie-und-arbeitspsychologie/gpower) based on a different dataset acquired in our lab, in which RTs were recorded during the very same VT paradigm employed here, in two different scenarios and a significant 2 × 3 interaction between scenarios and VT conditions (unimodal, bimodal near, bimodal far) was found. Since the common effect of interest with the present study was the difference between bimodal conditions in the two scenarios, the effect size (dz) was calculated on the delta between bimodal conditions in the two scenarios and a sample of 20 participants was estimated (α = 0.05; power (1 − β) = 0.85; dz = 0.63). Hence, 20 right-handed healthy subjects (﻿14 females, mean age ± SD = 27.45 ± 4.39, mean years of education ± SD = 18.05 ± 0.99) were recruited for the present study. All participants were naïve to the experimental procedure and gave informed consent to participate in the study. All the experimental protocols were approved by the Ethical Committee of the University of Turin (Comitato di Bioetica di Ateneo; protocol n° 133280) and were performed in accordance with the Declaration of Helsinki.

### Experimental procedures

**Figure 1:**
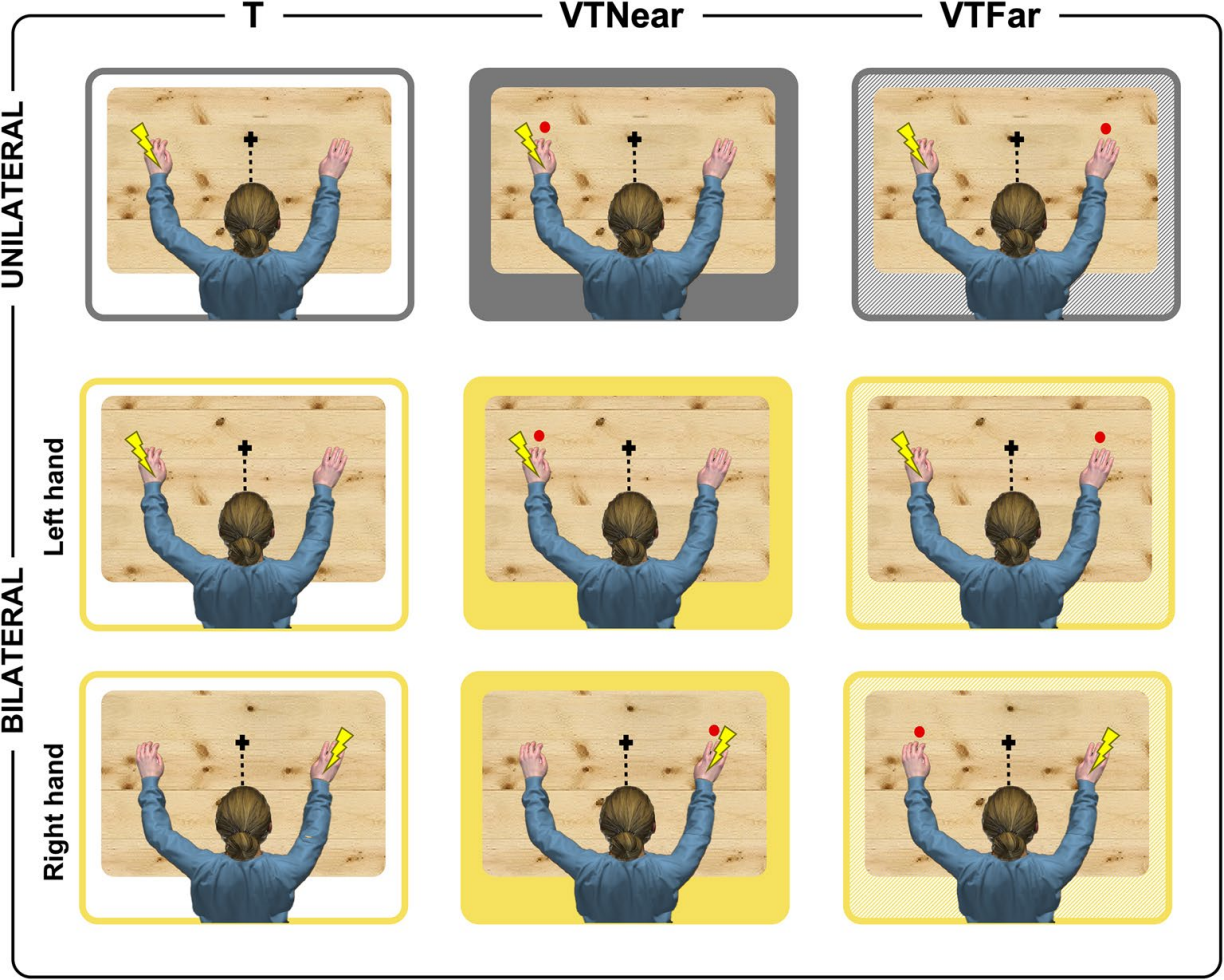
The figure shows a schematic representation of the experimental conditions (T, VTNear and VTFar) in the unilateral (grey) and bilateral (yellow) tasks. Red dots represent visual stimuli, while yellow thunders represent the tactile stimuli. Note that, in the bilateral task, tactile stimuli could occur either on the left or on the right hand with equal probability whereas, in the unilateral task, they always occurred on the left hand.

Participants comfortably sat at a desk with their hands laying at a distance of 40 cm from one another. Participants underwent two subsequent blocks, i.e., the unilateral and the bilateral task, in a counterbalanced order so that half of the participants started with the unilateral task, whereas the other half started with the bilateral task. In the *unilateral task*, tactile stimuli were delivered to their left hand, in isolation (*T condition*) or simultaneously with a visual stimulus, appearing either close to (*VTNear condition*) or far from (*VTFar condition*) the stimulated hand. Note that, differently from attentional cueing paradigms, wherein the task-irrelevant stimuli precede the task-relevant ones by 100–300 ms^29,30^, here visual and tactile stimuli were delivered simultaneously in bimodal (VT) conditions. Hence, since tactile afferent stimuli reach primary cortices before visual stimuli^31^, the latter could never represent an attentional cue for the tactile target. Unimodal visual stimuli were also presented and served as catch trials. See below for tactile and visual stimulation details. Participants were asked to gaze at a fixation cross aligned with their body midline (see Fig. 1) and to respond to tactile stimuli as fast as possible by pressing a pedal with their right foot, while ignoring the visual stimuli. After each trial, they also rated the perceived intensity of the tactile stimulus on a Likert scale (0–7), ranging from “no stimulus” to “the most intense tactile stimulus I can imagine”. The *bilateral task* was identical to the *unilateral task*, except that in the former tactile stimuli could occur with the same probability on either the right or the left hand. During both tasks, the stimuli for all conditions (i.e., T; VTNear; VTFar) were randomly presented, with a jittering inter-stimulus interval ranging from 4 to 5 s, into two blocks, including a total of 24 trials for each condition (plus 48 catch trials, i.e., 24 visual-only stimuli for each position—VNear and VFar). Stimuli presentation and RTs recording were controlled by a custom script combining Arduino (http://www.arduino.cc) and E-prime 2.0 (Psychology Software Tools, http://www.pstnet.com) software.

### Experimental stimuli

*Tactile stimuli* were transcutaneous electrical stimuli consisting in constant current square-wave pulses (DS7A, Digitimer) delivered to the hand dorsum by means of three surface cup electrodes. Stimulus duration was 200 μs. Stimulus intensity, adjusted according to participants’ sensitivity, corresponded to the individual perceptual threshold * 2. Before the task, individual right- and left-hand thresholds were estimated, employing the method of limits^32,33^. Note that, in order to minimize habituation, stimuli were randomly delivered to two different stimulation sites on each hand, as in previous studies (Fossataro et al., 2020^8^; Galigani et al., 2020^9^). In any case, tactile electrical stimulation was never perceived as painful. The mean stimulus intensities ± SD were 3.66 ± 0.68 mA, range 2.8–5.2 mA for the right hand and 3.70 ± 0.68 mA, range 2.62–4.9 mA for the left hand.

*Visual stimuli* were brief flashes (50 ms duration) delivered through two red light emitting diodes (5 mm) mounted close to the stimulated portion of the participant’s hands (see Fig. 1).

### Data analysis

Data analyses were performed on the mean outlier-checked RTs and on the mean subjective ratings using Statistica Software (StatSoft, release 8). We considered as outliers the trials in which RTs exceeded two standard deviations below or above the mean (of each specific experimental condition), as well as the trials with missing response^8,34–37^. The average number of discarded responses per participant was around 5%. Normal distribution of the residuals was checked by means of Shapiro–Wilk test (RTs: p > 0.43; subjective ratings: p > 0.14).

For both *RTs* (performance analysis) and *subjective ratings* (perception analysis), the mean values were entered in a 2 × 3 repeated measures ANOVA with task (unilateral/bilateral) and condition (T/VTNear/VTFar) as within-subject factors. Post-hoc comparisons were performed with Bonferroni test. Note that significantly faster responses in the VTNear as compared to the VTFar condition indicate the presence of the space-dependent multisensory enhancement.

Here we analyzed only the responses to the left-hand stimuli (i.e., we excluded the responses to the right-hand stimuli of the bilateral task), in order to include the same number of trials per condition for each task and to exclude any stimulus–response compatibility confound ^38^. However, in the Supplementary Information, we performed some control analyses, comparing the responses to left-hand and right-hand stimuli, to rule out that significant differences emerged between stimulus responses on the two hands in the bilateral task.

Finally, following an anonymous reviewer’s suggestion, to normalize the values on a baseline, we performed a subtraction analysis using the unimodal condition, wherein the tactile stimulus is delivered without visual stimulation. Hence, separately for *RTs* and *subjective ratings*, we computed two multisensory facilitation indices for each task, as follows. For RTs we calculated a Near and a Far Index by subtracting the mean RTs of each bimodal condition to the mean RT of the unimodal condition (Index Near = T-VTNear; Index Far = T-VTFar). For subjective ratings we calculated a Near and Far Index by subtracting the mean RT of the unimodal condition to the mean RTs of each bimodal condition (Index Near = VTNear-T; Index Far = VTFar-T). Thus, for both measures, higher Index values indicated greater multisensory facilitation.

These indices were entered in two 2 × 2 repeated measures ANOVA with task (unilateral/bilateral) and condition (Near Index/Far Index) as within-subject factors. Post-hoc comparisons were performed with Bonferroni test.

## Results

### Performance analysis

**Figure 2:**
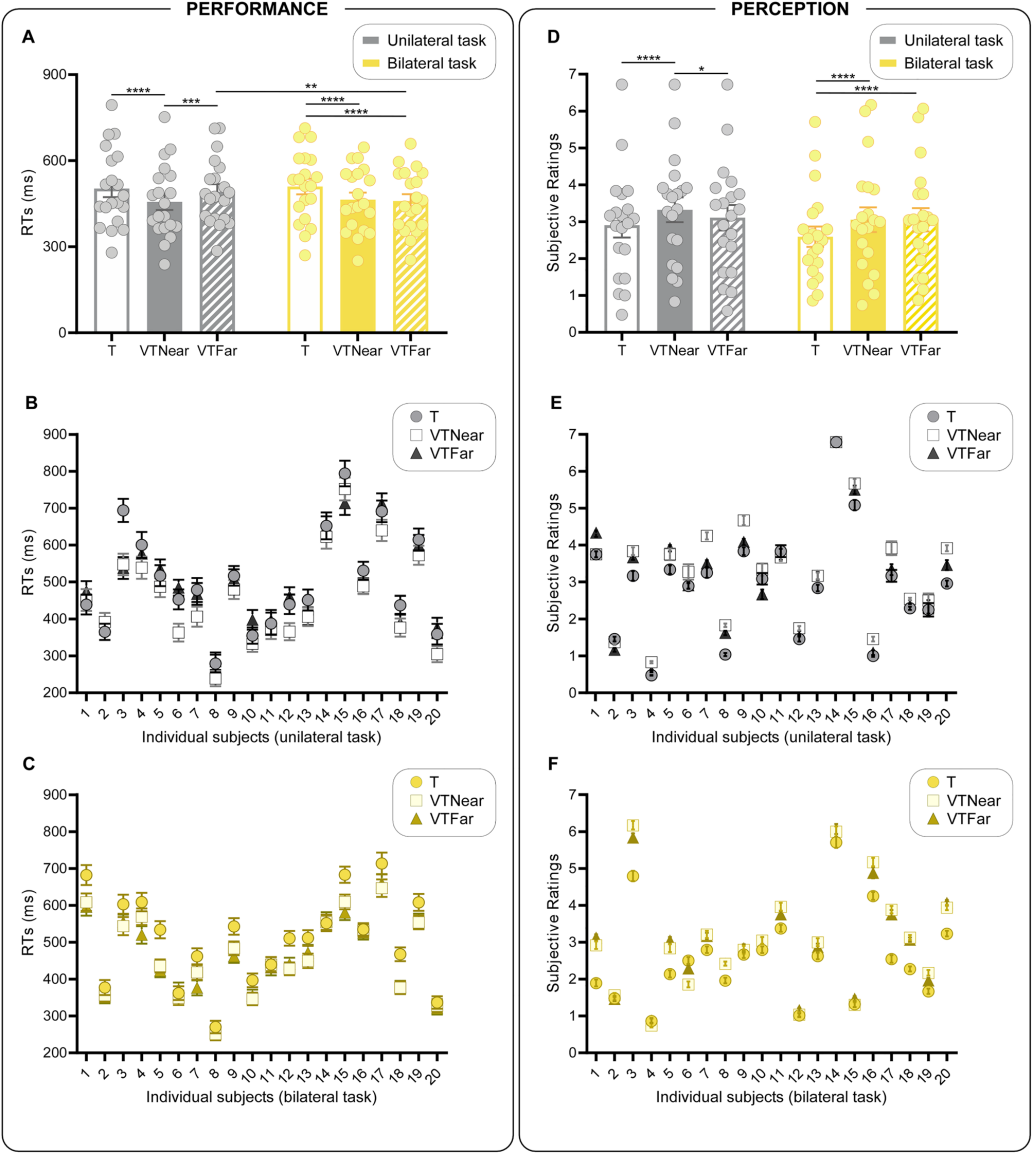
Performance and perception results. (**A,D)** Represent the task × condition interactions. Bars represent the mean RTs (**A**) and the mean subjective ratings (**D**) in T (empty), VTNear (solid), and VTFar (striped) conditions in the unilateral (grey) and in the bilateral (yellow) task. Error bars indicate standard error of the mean (SEM). Dots represent individual participants. *p < 0.05; **p < 0.01; ***p < 0.001; ****p < 0.0001. (**B,C,E,F)** Represent the mean RTs (**B,C**) and the mean subjective ratings (**E,F**) of each one of the 20 tested participants, in each condition, and separately for each task [unilateral: (**B,E**); bilateral: (**C,F**)]. Dots represent mean individual values in the T condition, squares represent mean individual values in the VTNear condition and triangles represent mean individual values in the VTFar condition. Error bars indicate standard error of the mean (SEM). Note that, while in the unilateral task the majority of participants show faster RTs and higher ratings in the VTNear as compared to both VTFar and T, in the bilateral task they show significantly slower RTs and lower ratings in T as compared to both VTFar and VTNear.

The 2 × 3 ANOVA on mean RTs highlighted a significant main effect of condition (F_2,38_ = 29.03; p < 0.0001; η^2^_p_ = 0.60), with faster responses in both the bimodal compared to the unimodal conditions (VTNear vs T: p < 0.0001, dz = 1.81; VTFar vs T: p < 0.0001, dz = 0.94), as well as faster RTs in the VTNear than in the VTFar condition (p = 0.049, dz = 0.65). Interestingly, a significant task × condition interaction was found (F_2,38_ = 9.79; p = 0.0004; η^2^_p_ = 0.34; see Fig. 2A). The Bonferroni post hoc test revealed that in the unilateral task the mean RTs were faster in the VTNear condition as compared to both the T (p < 0.0001, dz = 1.28) and the VTFar (p = 0.0004, dz = 0.92) conditions. Note that, in the unilateral task, the VTFar condition showed similar RTs to those of the T condition (p = 1, dz = 0.23). In the bilateral task, both bimodal conditions showed significantly faster RTs compared to the unimodal one (VTNear vs T: p < 0.0001, dz = 1.45; VTFar vs T: p < 0.0001, dz = 1.34), and, crucially, there was no difference between VTNear and VTFar conditions (p = 1.0, dz = 0.23). Finally, the comparison between the unilateral and the bilateral task’s VTFar conditions revealed slower RTs in the former compared to the latter (p = 0.001, dz = 0.51).

**Figure 3:**
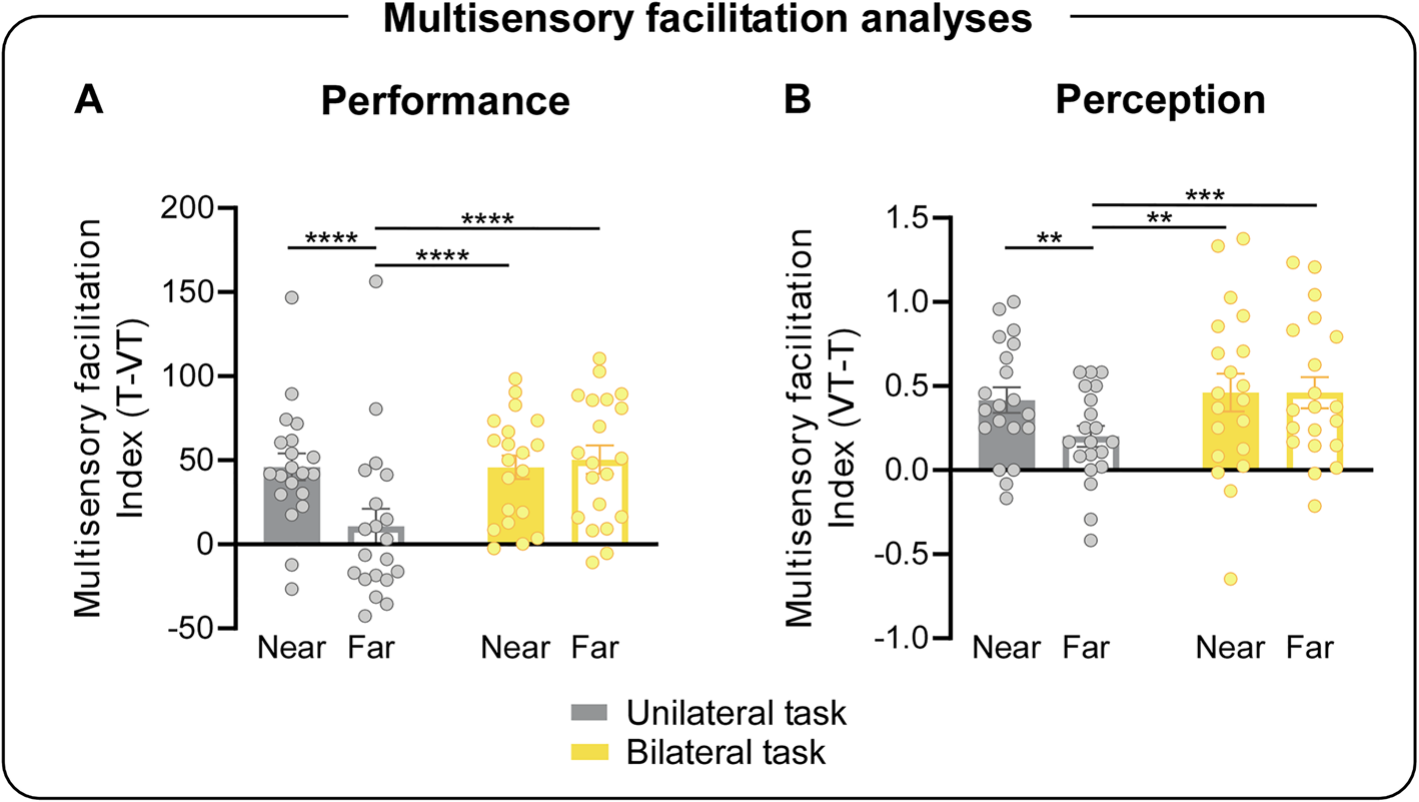
Multisensory facilitation analyses results, task × condition interaction. Bars represent the mean Multisensory facilitation Index [(**A**) *Performance*; (**B**) *Perception*] in Near and Far conditions. Note that, for both measures, higher values indicate greater multisensory facilitation. Error bars indicate standard error of the mean (SEM). Dots represent individual participants. ****p < 0.0001; ***p < 0.0005, **p < 0.005.

These findings were confirmed by the multisensory facilitation results. Indeed, the 2 × 2 ANOVA on Near and Far indexes revealed a significant main effect of condition (F_1,19_ = 8.52; p = 0.009; η^2^_p_ = 0.31), with greater multisensory facilitation in Near as compared to Far conditions. Crucially, a significant task × condition interaction (F_1,19_ = 21.22; p = 0.0002; η^2^_p_ = 0.53; see Fig. 3A) was found and Bonferroni post hoc tests showed that the multisensory facilitation was greater in Near as compared to Far condition, only in the unilateral task (p = 0.00009; dz = 0.91). Finally, the multisensory facilitation was greater in the Far condition of the bilateral task, as compared to that of the unilateral one (p = 0.00002; dz = 0.73).

### Perception analysis

The 2 × 3 ANOVA on the subjective ratings revealed a significant main effect of condition (F_1,19_ = 26.64; p < 0.0001; η^2^_p_ = 0.58), with significantly higher ratings in both the VTNear (p < 0.0001, dz = 1.36) and the VTFar (p < 0.0001, dz = 1.08) compared to the T condition. Crucially, a significant task × condition interaction was found (F_1,19_ = 4.14; p = 0.02; η^2^_p_ = 0.18; see Fig. 2D). The Bonferroni post hoc test revealed that in the unilateral task, mean ratings were higher in the VTNear condition as compared to both the T (p < 0.0001, dz = 1.22) and the VTFar (p = 0.047, dz = 0.69) conditions. Note that, in the unilateral task, the VTFar condition showed similar subjective ratings to those of the T condition (p = 0.08, dz = 0.71). In the bilateral task, both bimodal conditions showed significantly higher ratings compared to the unimodal one (VTNear vs T: p < 0.0001, dz = 0.92; VTFar vs T: p < 0.0001, dz = 1.11), but, crucially, there was no difference between VTNear and VTFar conditions (p = 1.0, dz = 0.002).

These findings were confirmed by the multisensory facilitation results. Indeed, the 2 × 2 ANOVA on Near and Far indexes revealed a main effect of condition (F_1,19_ = 6.22; p = 0.02; η^2^_p_ = 0.25), with higher multisensory facilitation in the Near as compared to the Far condition. Moreover, results revealed a significant task × condition interaction (F_1,19_ = 7.82; p = 0.01; η^2^_p_ = 0.29; see Fig. 3B), with greater multisensory facilitation in the Near as compared to the Far condition, only in the unilateral task (p = 0.005; dz = 0.67). Again, multisensory facilitation was greater in the Far condition of the bilateral task, as compared to that of the unilateral one (p = 0.0008; dz = 1.37).

## Discussion

The present study aims at providing a possible interpretation to the dissonant voices concerning the presence *vs* absence of a space-dependent multisensory integration effect. Here, we verified whether the presence of a space-dependent effect may be related to a saliency-driven mechanism, regulated by the probability of occurrence of tactile stimulation on different body districts. To this aim, we leveraged on a visuo-tactile (VT) paradigm^8^, in which tactile stimuli are delivered in isolation (unimodal) or simultaneously with visual ones, occurring either near to (bimodal near) or far from (bimodal far) the stimulated hand. This paradigm allows to measure possible space-dependent modulations of multisensory integration by comparing bimodal trials. In the unilateral task (where tactile stimulation had 100% probability to occur on the left hand), significantly faster responses were recorded when visual stimuli appeared in proximity of the stimulated left hand (i.e., within the left-hand PPS), as compared to when visual stimuli appeared in proximity of the non-stimulated hand (i.e., outside the left-hand PPS). Conversely, in the bilateral task (where tactile stimulation had the same probability—50%—to occur on both hands), no significant difference between bimodal near and bimodal far conditions was found (see Fig. 2). In other words, the occurrence of a visual stimulus in proximity of either the stimulated or the non-stimulated hand led to a comparable facilitation of the participants’ responses. Overall, our findings demonstrate that a space-dependent modulation of multisensory-driven facilitations was present only in the unilateral task, whereas it was abolished in the bilateral one.

It is important to note that, in previous studies using unilateral tasks similar to that employed here, auditory/visual stimuli occurring far away from the stimulated hand were often presented in an empty portion of space^8–14^. Conversely, auditory/visual stimuli always appeared in proximity of the non-stimulated hand in studies employing bilateral tasks^21–24,26^. Since non-informative vision of the body enhances tactile detection and multisensory integration^39,40^, it was not possible to exclude that the divergent findings concerning the presence/absence of space-dependent modulations could be explained by the simple presence of the non-stimulated hand close to the auditory/visual concurrent stimuli. In the present study, visual stimuli were always displayed near to the non-stimulated hand in the bimodal far conditions, both in the unilateral and in the bilateral task. Therefore, the presence of the hand alone cannot represent the main determinant of our findings.

**Figure 4:**
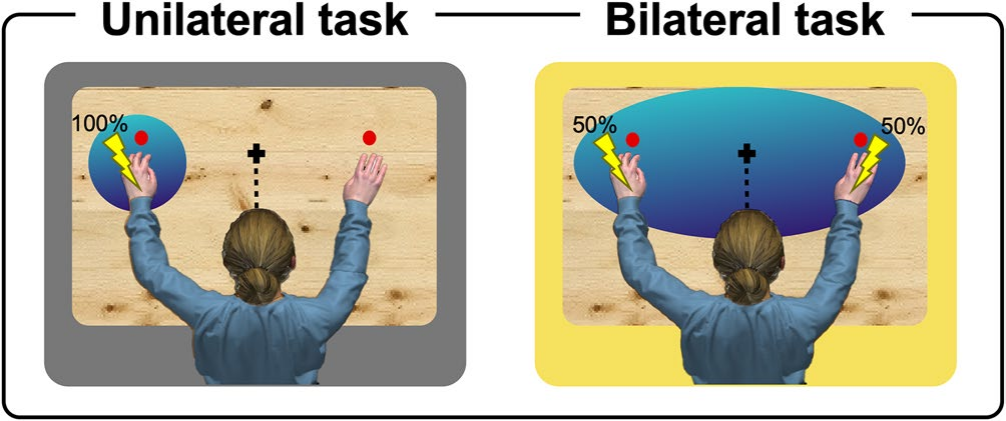
PPS plastic changes. The results suggest that the PPS boundaries are shaped according to the saliency afforded to each hand. When the probability of receiving a tactile stimulus is 100% on the left hand and 0% on the right hand, the PPS is limited to the space immediately surrounding the left hand. Conversely, when the probability of receiving a tactile stimulus is equally distributed between the two hands (50% on each hand), the PPS seems to enlarge as to include the space surrounding both hands.

Here, the differential findings between the two tasks were driven by the responses in bimodal conditions, which resulted faster in the bimodal near than in the bimodal far condition in the unilateral task, whereas such a difference was absent in the bilateral task. In other words, visual stimuli occurring in the same portion of space were coded as within or outside the PPS (as indexed by the presence *vs* absence of the corresponding multisensory facilitation) depending on the probability of receiving a tactile stimulus on each hand. This finding suggests that the brain representation of the PPS around the hands is updated, from time to time, on the basis of an automatic detection of the environmental sensory regularities occurring around the body. According to our interpretation, the spatial-dependent multisensory enhancement is modulated by the expectations induced by the predictability of tactile stimulation on different body parts. Then, the PPS boundaries are defined according to the estimated saliency of the sensory events occurring in the space surrounding each body part in the specific context. Indeed, in the unilateral task, wherein the probability of occurrence of tactile stimulation was 100% on the left hand and 0% on the right hand, visual stimuli occurring close to the right hand induced slower responses, thus indicating that they were coded as outside the PPS. Conversely, in the bilateral task, wherein the probability was equally distributed between the two hands (50% on each one), visual stimuli occurring close to the right hand produced faster responses (irrespective of whether the tactile stimulation was delivered on the right hand or on the left hand in that specific trial), thus showing that they were coded as within the PPS. In other words, anytime the nervous system may produce a reliable estimation of which body district will be stimulated, the PPS is limited to the space immediately surrounding such body district. When an estimation cannot be made (because the probability of occurrence of incoming stimulus location is at chance), it seems that the nervous system adopts a more conservative strategy, by extending the PPS to include each potentially-stimulated body district (see Fig. 4). From an evolutionary point of view, this conservative choice, by enhancing the saliency of each potentially-involved body district, allows the individual to best detect and react to any incoming sensory stimulation.

Note that, the abolishment of the spatial modulation of multisensory integration (i.e., the absence of a difference between VTNear and VTFar) found in the bilateral task could have two explanations. First, it could be the consequence of an *expansion* of the PPS. If the “far” space is remapped as “near”, the multisensory facilitation is the same for both spaces, so no difference between VTNear and VTFar is observed. Previous evidence already showed that the PPS representation can be plastically expanded following visuo-motor trainings^9,18,41^ or multisensory illusions^42^, so that what was previously coded as “far” can become “near”, being included within the PPS. Second, the absence of the spatial modulation of multisensory integration could be the result of an *abolishment* of the PPS. If the “near” space is no longer treated as a special portion of space, the multisensory facilitation is abolished in the “near” space, therefore no difference between VTNear and VTFar is observed.

The present data might help in disentangling between these two interpretations. If the “far” space became “near” in the bilateral task, the same multisensory enhancement in VTNear and VTFar should have been observed, indexed by a significant enhancement of the responses in both bimodal conditions as compared to the unimodal (T) condition. In this case, the absence of a difference between bimodal conditions in the bilateral task is explained by the enhancement of the responses in the VTFar condition, which become similar to those of the VTNear condition. Conversely, if the “near” space was no longer treated as a special portion of space and PPS was abolished in the bilateral task, we should have observed the annulment of the multisensory enhancement in both bimodal conditions, indexed by the absence of the difference between bimodal conditions and the unimodal one.

Our data support the *expansion* hypothesis, showing enhanced responses (not significantly different from VTNear) in the VTFar of the bilateral task, as if this portion of space was recoded as “near” space, when the sensory environment was unpredictable. However, we must acknowledge that with the present data we cannot conclude whether the expansion is limited to the space surrounding each hand or whether it also includes the space in between the hands.

Supporting evidence to the present results comes from previous research investigating the hand-blink reflex (HBR), an automatic defensive mechanism. In a very elegant study, Sambo and colleagues^43^ observed a modulation of the defensive PPS boundaries depending on the probability of a threatening stimulus occurrence in different locations in space. It is well known that the HBR, elicited by the stimulation of the wrist median-nerve, is enhanced when the stimulated hand is near to the head^43–46^. In Sambo and colleagues^43^, the authors demonstrated that the HBR enhancement was present when the stimuli were delivered over the hand entering the head PPS. Conversely, when stimulations occurred always on the non-moving hand which was kept far from the head, no enhancement was measured. Interestingly, when the probability of receiving a stimulus was equally distributed between the hands (i.e., the hand entering the head PPS or the static hand which was always kept far from the head), the HBR enhancement was elicited by the stimulation of both hands^43^. Overall, these findings seem to confirm the presence of an automatic mechanism controlled by environmental sensory regularities, able to dynamically adjust the extent of the PPS, either when functioning as a safety margin surrounding the body [defensive PPS^3,47,48^], or as the preferential space for multisensory integration ^1^. In line with this view, a recent neurophysiological study by Kosciessa et al., similarly pointed out that the brain actively regulates the processing of complex inputs according to contextual uncertainty, generalizing sensory gain enhancements with increasing uncertainty^49^.

Influential views in cognitive neuroscience postulated that attention may be the medium through which a flexible body representation is implemented in the brain^50^. In other words, the modulation of the PPS extent should be mediated by a reorienting of attention, induced by environmental sensory regularities. Nevertheless, other studies previously showed that PPS reshaping is independent from attentional modulations^9,18^. Even though attentional re-orienting may be associated to PPS reshaping, it is unlikely that attention alone could explain the present findings. Works on endogenous spatial attention showed that attention grants an improvement in the overall performance in tactile tasks^51^. Therefore, an attentional modulation in our tasks should have enhanced the responses to the tactile stimuli in the unilateral task (where attention can be focused on one single hand) as compared to the bilateral task (where the attentional focus is divided between the two hands). However, this is not the case, since, in our data, responses to unimodal tactile stimuli did not differ between unilateral and bilateral tasks. The results show a significant difference between tasks only in the VTFar condition, suggesting that that the very same bimodal stimulation (i.e., VTFar, wherein the visual stimulus occurred on the opposite side as compared to the tactilely stimulated hand) was coded differently depending on the probability of stimulus occurrence. This finding supports the hypothesis that PPS reshaping may also occur independently from attentional modulations.

Moreover, our results, showing a space-dependent multisensory enhancement in the unilateral but not in the bilateral task, can also be observed at the explicit level, measuring the perceived intensity of tactile stimuli reported by the participants. In the unilateral task, the intensity of tactile stimuli was perceived as higher when the concomitant visual stimulus was presented near to the stimulated hand (i.e., bimodal near condition), as compared to the bimodal far and the unimodal conditions. Conversely, in the bilateral task, participants reported higher intensity ratings in both bimodal conditions, as compared to the unimodal one, regardless of the visual stimulus location. These findings suggest that multisensory integration processes and their space-dependent modulations are captured not only by low-level measures of brain reactivity (such as RTs), but also by explicit perceptual judgements. Even though a pioneering study highlighted that multisensory-driven facilitation may result in enhanced perceived intensity during an audio-visual task^52^, to our knowledge, the present study represents the first evidence that the subjective perceived intensity of the incoming multisensory stimulation is shaped by the proximity to the body. Given the complete paralleling between the RT and the subjective rating results, we speculate that the latter may represent the conscious feedback of the implicit phenomenon indexed by the former. From an evolutionary perspective, the presence of a conscious correlate of the implicit mechanism described above may have the function of further enhancing the saliency of the sensory events in the space surrounding the body, thus contributing to the control of intentional motor actions, crucial to realize coordinated defensive or interacting behaviors.

## Conclusion

Taken together, our findings suggest that an automatic detection of sensory regularities modulates multisensory responses within the PPS. This represents a theoretical advance in the field of experimental psychology and cognitive neuroscience, able to bridge the gap between previous divergent findings concerning the interpretation of space dependent multisensory integration effects. Future studies should be devoted to further investigate the mechanism uncovered by the present research, providing additional insights on its functioning, such as exploring whether, when PPS is enlarged due to sensory unpredictability, the expansion is limited to the space surrounding each hand or whether it also includes the space in between the hands. This issue could be addressed by employing a looming multisensory paradigm, wherein different distances from the body are investigated (e.g., see^13^). Finally, future studies could be directed to highlight a neurophysiological correlate of the observed behavioral effects.

## Supplementary Information

Supplementary Information.

## Data Availability

Data and materials for all experiments are available here.
