# Supplementary material for: Multisensory-driven facilitation within the peripersonal space is modulated by the expectations about stimulus location on the body
Source: Sci Rep. 2022 Nov 21;12:20061. doi: 10.1038/s41598-022-21469-w (PMC9681840; doi:10.1038/s41598-022-21469-w)
Supplement: Supplementary file 1 — Supplementary Information. [file 41598_2022_21469_MOESM1_ESM.docx]

**Supplementary Information**

**Multisensory-driven facilitation within the peripersonal space is modulated by the expectations about stimulus location on the body**

**Alice Rossi Sebastiano, Irene Ronga, Carlotta Fossataro, Mattia Galigani, Karol Poles, Francesca Garbarini**

**Additional data analyses**

To rule out that significant differences emerged between the left- and the right-hand stimuli responses in the bilateral task, we performed the following additional analyses. For both *RTs* (performance analysis) and *subjective ratings* (perception analysis), the mean values were entered in a 2﻿×3 repeated measures ANOVA with hand (right/left) and condition (T/VTNear/VTFar) as within-subjects factors. Post-hoc comparisons were performed by means of Bonferroni test.

**Additional analyses results**

**Performance analyses.** The 2×3 ANOVA on mean RTs highlighted a significant main effect of condition (F_2,38_=51.14; p<.0001; η^2^ _p_=.73; see Figure S1), with faster responses in both the bimodal compared to the unimodal conditions (VTNear vs T: p<.0001, dz=1.97; VTFar vs T: p<.0001, dz=1.48), whereas no difference emerged between VTNear and VTFar conditions (p=1.0, dz=0.25). Importantly, no significant main effect of hand nor hand × condition interaction was found, confirming that there was no difference between the right- and the left-hand responses in the bilateral task.

**Perception analyses.** Coherently, the 2x3 ANOVA on the subjective ratings revealed a significant main effect of condition (F_1,19_=18.83; p<.0001; η^2^ _p_=.50; see Figure S1B), with significantly higher ratings in both the VTNear (p<.0001, dz=1.06) and the VTFar (p<.0001, dz=0.91) compared to the T condition, whereas no difference emerged between VTNear and VTFar conditions (p=1.0; dz=0.54). Again, no significant main effect of hand nor hand × condition interaction was found for both RTs and subjective ratings.


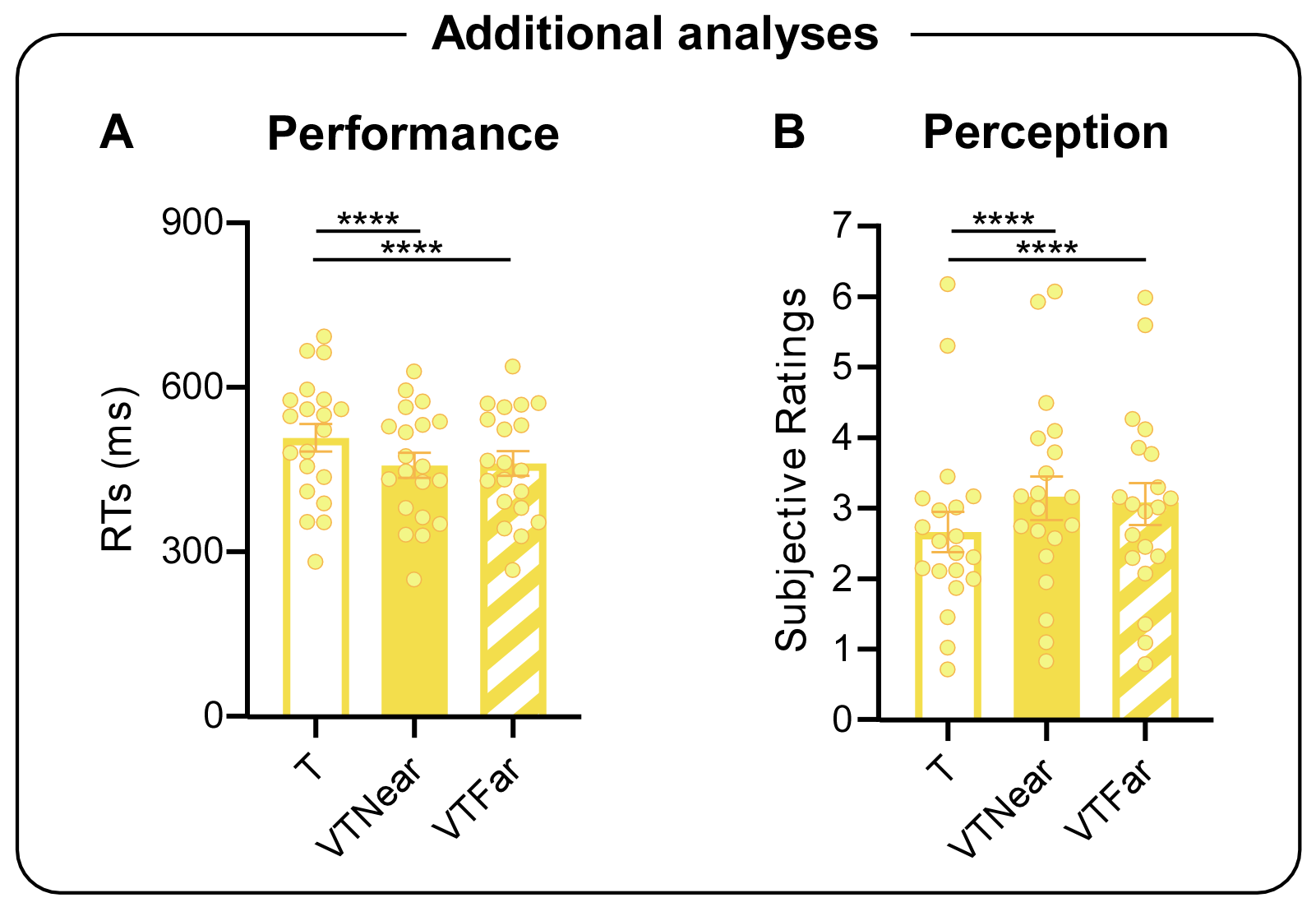


**Figure S1.** Additional analyses results, main effect of condition. ﻿Bars represent the mean RTs (panel A *Performance*) and the mean subjective ratings (panel B *Perception*) in T (empty), VTNear (solid), and VTFar (striped) conditions, irrespective of the stimulated hand. Note that, in the additional analyses, responses to right- and left-hand stimuli of the bilateral task were considered. Error bars indicate standard error of the mean (SEM). Dots represent individual participants. ****p<0.0001.
